# Experimental therapeutic assays of *Tephrosia vogelii* against *Leishmania major* infection in murine model: in vitro and in vivo

**DOI:** 10.1186/s13104-017-3022-x

**Published:** 2017-12-06

**Authors:** Sylvia Naliaka Marango, Christopher Khayeka-Wandabwa, Judith Alice Makwali, Bernard Ngoitsi Jumba, Joseph K. Choge, Eric Onyango Adino, Christopher O. Anjili

**Affiliations:** 1 Department of Biological Science (Parasitology), University of Eldoret, P.O Box 1125, Eldoret, 30100 Kenya; 2 School of Pharmaceutical Science and Technology (SPST), Health Sciences Platform, Tianjin University, Tianjin, 300072 China; 3 African Population and Health Research Center (APHRC), P.O. Box 10787, Nairobi, 00100 Kenya; 4 Centre for Biotechnology Research and Development (CBRD), Kenya Medical Research Institute (KEMRI), P.O Box 54840, Nairobi, 00200 Kenya; 5 Applied Science Department, Sigalagala National Polytechnic, Kakamega, Kenya; 6 Department of Medical Laboratory Sciences, MasindeMuliro University of Science and Technology, Kakamega, Kenya; 7 University of Kabianga, P.O. Box 2030, Kericho, 20200 Kenya; 8 East African Breweries Limited, Nairobi, Kenya

**Keywords:** Leishmaniasis, *Leishmania major*, *Tephrosia vogelii*, Pentostam, Amphotericin B, 50% inhibitory concentration, Toxicity, Efficacy

## Abstract

**Background:**

Conventional targeted leishmanicidal chemotherapy has persistently remained prohibitive for most economically deprived communities due to costs, associated time to accessing health services and duration for successful treatment programme. Alternatives are bound to be incorporated in rational management of leishmaniasis by choice or default due to accessibility and cultural beliefs. Therefore, there is need to rigorously investigate and appraise the activity of medicinal compounds that may have anti-leishmanicidal activity especially in the context of products that are already being utilized by the populations for other ailments but have limited information on their therapeutic value and possible cytoxicity. Hence, the study examined both in vivo and in vitro response of *L. major* infection to *Tephrosia vogelii* extracts in BALB/c mice as the mouse model.

**Methods:**

A comparative study design was applied for the in vivo and in vitro assays of the extract with Pentostam (GlaxoSmithKline, UK) and Amphotericin B [Fungizone™, X-Gen Pharmaceuticals (US)] as standard drugs.

**Results:**

In BALB/c mice where the chemotherapeutic extract was administered intraperitoneally, there was significantly (*p* < 0.05) larger reduction in lesion size and optimal control of parasite burden than those treated orally. However, standard drugs showed better activity. *Tephrosia vogelii* had 50% inhibitory concentration (IC_50_) and IC_90_ of 12 and 68.5 μg/ml respectively, while the standard drugs had IC_50_ and IC_90_ of 5.5 and 18 μg/ml for Pentostam and 7.8 and 25.5 μg/ml for Amphotericin B in that order. In the amastigote assay, the infection rates decreased with increase in chemotherapeutic concentration. The multiplication indices for *L. major* amastigotes in macrophages treated with 200 µg/ml of the standard drugs and extract were significantly different (*p* < 0.05). 200 µg/ml of *T. vogelii* extract showed a multiplication index of 20.57, 5.65% for Amphotericin B and 9.56% for Pentostam. There was also significant difference (*p* < 0.05) in levels of Nitric oxide produced in the macrophages.

**Conclusions:**

The findings demonstrated that *T. vogelii* extract has anti-leishmanial activity and further assays should be done to ascertain the active compounds responsible for anti-leishmanial activity.

## Background

The various forms of leishmaniases are considered neglected tropical diseases (NTDs). They affect majorly the economically disenfranchised populations in sub-Saharan Africa where, most of the affected do not seek and/or often synergize conventional medical attention with complementary options to alienate associated chronic suffering and opportunistic infections risks [1–4]. The observed trend has been linked to rational treatment and control challenges which include but not limited to prolonged period of time to fulfill standard chemotherapy regimen, associated costs, considerable level of side effects and recently, the emergence of antimony-resistant *Leishmania* strains in some endemic areas [4–8]. Equally, individually held knowledge by the traditional health practitioners (THP) has demonstrated a lot of complementary medicine diversity. This knowledge ranges from medicinal flora that can cure a variety of diseases with valuable clues for potentially anti-parasitic and protozoa compounds as well as current trends in terms of milestones, challenges and opportunities [3, 9–11].

Like most other settings globally, Kenya has a diversity of ethnicities and each has its own peculiar customs and beliefs and still rely on herbal remedies and trust the inherent potency value even when they can access modern medicine regardless of being rural or urban [12–14]. More than often, choosing to combine both herbal and modern medicine because of the belief they can target more than one complication at ago [13, 14]. Despite this understanding, most of the complementary medicines have not been documented and scientifically evaluated at fundamental assay level to determine their efficacy and dosage vis-à-vis the alleged diverse synergistic benefit of acting on a wide range of possible infections as often advanced by the Traditional health practitioners and communal history that vary by ethnicities [4, 14–16].

Various species from the family Fabaceae have demonstrated a diversity of applicability as chemotheraputants. Extracts of the leaf, bark, root, seed and flower (with literature suggesting predominance focus on the leaves) have demonstrated anti-leishmanial, antimicrobial, anthelmintic, antiprotozoal, analgesic, antioxidant and anti-inflammatory activity when tested in vitro and/or in vivo in different animal and murine models [17–22] while vector control worth and mitigation of various intermediate hosts (e.g. larvicidal activity against *Aedesaegypti,* Acaricidal activity on ticks, *Biomphalariapfeifferi* and *Bulinustruncatus*) have also been reported widely [20, 23–25]. *Tephrosia purpurea* (family: Fabaceae), commonly used in traditional remedies for the treatment of febrile attacks, enlargement and obstruction of liver, spleen, and kidney, was found to have significant antileishmanial activity, and has been evaluated extensively in various experimental animal models to locate the abode of activity [22] with near nil evidence from Kenya on the same or related family and species. The leguminous *Tephrosia vogelii* from the same family Fabaceae and class Magnoliopsida (dicotyledons), subclass Rosidae order Fabales, has been and is being used extensively in Kenya to improve soil fertility and as a medicinal product against various antimicrobial, protozoa and parasitic infections [16, 21, 26, 27]. Preferential and selective validation of the medicinal properties of the plant has resulted to progressively limited understanding on anti-protozoa capacity of the plant and as to whether the leguminous extracts would also be having anti-leishmanicidal activity with the understanding of the fact that leishmaniases are caused by obligate intracellular kinetoplastid protozoa of the genus *Leishmania*. Equally, beyond the scope of this study, the in vitro investigation enhances the availability of information on possible extent of the extract cytoxicity and safety in the Kenyan context where the information is sparse. In consideration of the ascribed available circumstantial animal and murine experimental evidence, gaps and envisaged information benefit in the context of the tropical forested ecological and ethno botanical diversity for alternative medicinal compounds indepth understanding and precisely *Tephrosia vogelii* [4, 7, 10, 28, 29], the aim of this study was to assess the response of *Leishmania major* to *Tephrosia vogelii* therapy in BALB/c mice (in vivo) and also in vitro in a quest to shape and consolidate baseline evidence on cytotoxicity and activity in the home-grown context and pave way for indepth validation.

## Methods

### Experimental setup

The in vivo and in vitro studies were carried out using a comparative study design. The efficacy and toxicity of the sample was compared with those of Pentostam (GlaxoSmithKline, UK) and Amphotericin B [Fungizone™, X-Gen Pharmaceuticals (US)]. Rosewell Park Memorial Institute 1640 Medium (RPMI) was used as control in in vitro experimental chemotherapeutic studies. In vivo studies were further subjected to a complete randomized block design. Phosphate Buffered Saline was used as the control. The results were compared to determine the efficacy of the test sample against the known standard drugs for treating leishmaniasis.

### Plant collection and extract preparation

Identified and dried *T. vogelii* leaves were obtained from Kenya Medical Research Institute (KEMRI) herbarium, Nairobi. Extraction was done in KEMRI, Nairobi Kenya at the Centre for Traditional Medicine and Drug Research (CTMDR). The dried leaves were processed (to obtain the extract) as earlier described by Kigondu et al. [28]. The dry leaves were chopped into small pieces and then ground into powder form using a laboratory blender. 1 kg of each powder was soaked in absolute methanol for 3 days to extract compounds. The extract was filtered, dried with Na_2_SO_4_ and the solvent removed under vacuum in a rotary evaporator at 30–35 °C and stored at 20 °C until required for use. This methanolic extracts were used for anti-leishmanial tests.

### Mice, *Leishmania* parasites and experimental infections

The BALB/c female mice aged 8 weeks and weighing 20  ±  2 g used for the study were obtained from the KEMRI’s animal house facility in Nairobi. The animals were moved into the experimental room for acclimatization 1 week before the start of the experiments. The mice were housed in 15 cm × 21 cm × 29 cm transparent cages. They were fed with pellets (Mice pellets UNGA^®^ feeds) and water ad libitum.

*Leishmania major* (strain IDUB/KE/83=NLB-144) which was originally isolated in 1983 from a female *Phlebotomus dubosqi* collected near marigat, Baringo County in Kenya were used [30]. These parasites were cultivated in schneider’s Insect Medium supplemented with 20% heat inactivated foetal bovine serum, 100 µg/ml penicillin and 50 µg/ml streptomycin and 250 µg/ml 5-fluorocytosine arabinioside [31, 32]. This strain has been maintained by cryopreservation and in vitro culture and periodic passage in BALB/c mice at KEMRI, Nairobi. Promastigotes were incubated at 25 °C grown to stationary phase to generate infective metacyclic forms at 6th day of culture. Promastigotes in the medium were counted with a hemocytometer (Improved Double Neubauer) (Pharmacia-GE Healthcare, Uppsala, Sweden) with a Nikon optiphot optical microscope at 40× magnification.

### In vitro studies

#### Cytotoxicity assays

Vero cells were cultured and maintained in Minimum Essential Medium (MEM) supplemented with 10% Foetal Bovine Serum (FBS). The cells were cultured at 37 °C in 55% CO_2_ for 24 h, harvested by trypsinization, pooled in a 50 ml vial and 100 µl cell suspension (1 × 10^6^ cells/ml) put into 2 wells of rows A-H in a 96-well micro titer plate, the medium aspirated off and 150 µl of the highest concentration (1000 µg/ml) of the *T. vogelii* added into the first row and serial dilution carried out. The experimental plates were incubated further at 37 °C for 48 h. The controls used were cells with no extract but medium alone. MTT reagent (10 µl) was added into each well and the cells incubated for 4 h until a purple precipitate clearly visible under a microscope was formed. The medium together with MTT were aspirated off from the wells. Dimethylsulfoxide (DMSO) (100 µl) was added and the plates shaken for 5 min. The absorbance was measured for each well at 562 nm using a micro-titre plate reader [33].

#### Evaluation of IC_50_ concentration and anti-promastigote assay

*Leishmania major* promastigotes (10^6^ parasites/ml) were incubated at 26 °C for 120 h in fresh media (brain heart infusion medium), Supplemented with 10% FBS in the absence or presence of serial concentrations (100, 50, 25, 12.5, 6.25 and 3.125 µg/ml) of the extracts (cell growth was determined daily by assessment of visible turbidity). The IC_50_ was considered as the concentration that inhibited 50% of *L. major* growth in vitro*. Leishmania major* promastigotes were incubated in 24-well plates in the presence of different concentrations of the extracts added. After 5 days of cultivation, aliquots of parasites were transferred to a 96-well micro-titre plate. The parasites were then incubated at 27 °C in 5% CO_2_ for 24 h; 200 µl of highest concentration of extract was added and diluted. The experimental plates were incubated further at 27 °C for 48 h. The controls used were promastigotes with no extracts and medium alone. In each well, 100 µl of DMSO was added and the plates shaken for 5 min. Absorbance was measured for each well at 562 nm using a micro titre reader [34].

#### Anti-amastigote assay

Peritoneal macrophages were harvested from 8 week old female BALB/c mice using Phosphate Buffered Saline 4 days after intraperitoneal injection at the Kenya Medical Research Institute (KEMRI) animal breeding facility, Nairobi-Kenya. After harvesting, the macrophages were processed as earlier described [35]. The obtained macrophages were then infected with *L. major* stationary phase promastigotes at a 6:1 parasite/macrophage ratio. The infected macrophages were incubated at 37 °C in 5% CO_2_ for 4 h. After incubation the remaining parasites were washed off using PBS and the cultures incubated in RPMI for 24 h. Treatment of infected macrophages with the experimental chemotheraputants was done once. Pentostam and Amphotericin B were used as standard treatment control drugs for comparison of parasite inhibition. The medium and drug were replenished daily for 3 days. On the 4th day, the monolayers were washed with PBS at 37 °C, fixed in methanol and stained with Giemsa. The number of amastigotes was determined by counting at least 100 macrophages in duplicate cultures, and the results expressed as infection rate (IR) and multiplication index (MI) as well as IC_90_ and IC_50_ with varied concentration rates of test drugs being 25, 50, 100 and 200 µg/ml [36–39].

#### Nitric oxide production assay

Nitric oxide release in macrophage cultures was measured using the Griess reaction for nitrites [40]. Supernatants were collected 48 h after introducing the test drug into the culture medium (in the amastigote assay) and stored at − 70 °C for analysis of nitric oxide (NO) production. Nitrite (NO_2_) accumulation in the cell culture supernatants was used as an indicator of nitric oxide production and it was determined by a standard Griess reaction [41–43].

### In vivo studies

#### Mice and parasite inoculation, quantifying parasite burden from spleen and liver

BALB/c mice were inoculated with 1 × 10^6^ stationary phase *L. major* promastigotes in 50 μl phosphate buffered saline into the Left Hind Footpad (LHFP) using a 29 gauge needle and left for 4 weeks incubation period [41, 44]. The inoculated mice were then randomly assigned into 5 groups of 8 mice in each. Group 1 was treated with *T. vogelii* extracts orally (0.2 mg/ml [200 µg/ml]), group 2 with *T. vogelii* extracts intraperitoneally (0.2 mg/ml [200 µg/ml]), group 3 with Pentostam intraperitoneally, group 4 with Amphotericin B intraperitoneally and group 5 with sterile PBS intraperitoneally at 100 µg/ml in the order for the latter three while PBS was used as the solvent-treatment commenced at the start of the 5th week post infection (day 29), for 28 days [44–47]. Lesion sizes (the difference in thickness between the infected footpad and the non-infected contralateral Footpad) were monitored weekly up to week 10; by measurement using a Starret dial caliper (Mitutoyo, Suzano, SP, and Brazil) [44, 48, 49]. The weight of the mice was also monitored on a weekly basis. A total of four randomly selected mice per group were sampled at week 10 post infection and chemotherapy for analysis of *L. major* parasite loads. The mice were anesthetized intraperitoneally with Pentobarbital sodium 80 mg/kg (Rompun; Bayer Plc., Newbury, UK). The liver and spleen were removed and weighed and changes post-infection and chemotherapy were determined based spleno-somatic and hepato-somatic indices [44]. Splenic and liver *L. major* burdens were determined from Giemsa-stained impression smears and expressed as Leishman-Donovan units (the number of amastigotes per 1000 host nuclei, multiplied by the weight of the organ) [43, 44].

#### Data analysis

The data collected on lesion sizes, parasite loads and absorbance were analyzed using the SPSS software. All experiments were performed in triplicate. The mean and standard errors of the lesions of each treatment group were compared using students’ t test and analysis of variance (ANOVA). Students’ t test was used in the analysis of differences in means obtained for the experimental groups. Chi square test was used in the analysis of infection rates and multiplication indices. p values of equal or less than 0.05 (*p* ≤ 0.05) were considered significant.

## Results

### Cytotoxicity assay

**Fig. 1:**
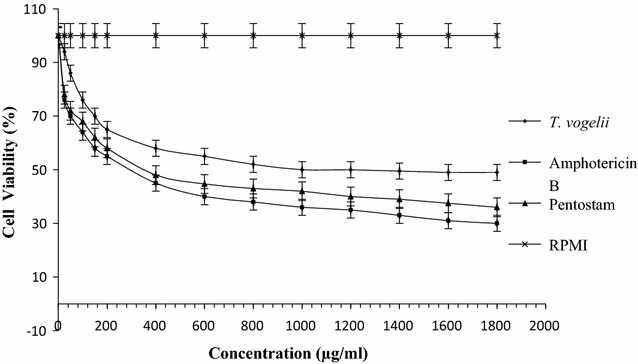
Cell viability of the vero cells subjected to the different test drugs

Results indicating the cell viability of vero cells subjected to the test compounds are shown in Fig. 1. The safety of the test extract was evaluated by treating vero cells with the extracts and viability was determined. Crude extracts of *T. vogelii* caused no significant adverse effects on the vero cells. Concentration of the standard drugs and test extract required to destroy 50% of the mammalian cell was significantly low in Amphotericin B (200 µg/ml), followed by Pentostam (250 µg/ml) and *T. vogelii* (1200 µg/ml). RPMI served as the control hence did not have any effect on the vero cells. Minimum inhibitory concentration for all the test drugs did not result in significant vero cell destruction. For Amphotericin B 1% of the cells were destroyed with 10 µg/ml concentration, Pentostam 1% were destroyed with 12 µg/ml treatment while for *T. vogelii* 1% were destroyed with 25 µg/ml treatment.

### Efficacy of *T. vogelii* crude extracts on promastigotes of *L. major*

The promastigote growth inhibition was significantly affected by the various experimental drugs (*p* < 0.05) after 24 h of exposure. The optimal efficacy of the standard drugs was 97.5 and 98.9% for Amphotericin B and Pentostam respectively. *Tephrosia vogelii* achieved an optimal efficacy of 91.4% against promastigotes. There was a significant difference in the IC_50_ and IC_90_ of the test compounds (*p* < 0.05). The standard drugs were more effective against promastigote as compared to *T. vogelii* with Pentostam having an IC_50_ of 5.5 μg/ml, Amphotericin B with 7.8 μg/ml while that of *T. vogelii* being 12 μg/ml. There was significant (*p* < 0.05) difference in the IC_90_ with the lowest IC_90_ occurring in Pentostam followed by Amphotericin B then by *T. vogelii* with 18, 25.5 and 64 μg/ml respectively. Table 1 describes the optimal efficacy, IC_90_ and IC_50_ of the test drugs against promastigote forms of the parasites.

**Table 1:** Optimal efficacy, IC_90_ and IC_50_ of test drugs against promastigote form of the parasites

Concentration (μg/ml)	Test drugs			Parameter and statistics	
	*T. vogelii*	Pentostam	Amphotericin B	F value	p value
Optimal efficacy (%)	92.0	98.9	97.5	36.654	0.0001
IC_90_	64	18.0	25.5	65.226	0.0002
IC_50_	12.0	5.5	7.8	85.456	0.001

### Efficacy of *T. vogelii* extract on amastigotes of *L. major*

**Fig. 2:**
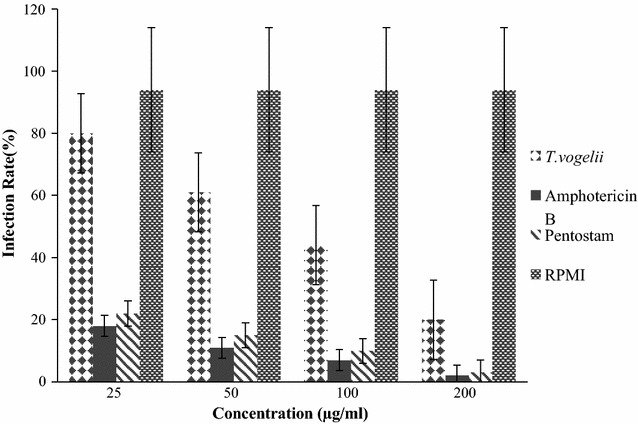
The infection rates of amastigotes in macrophages following treatments with various test drugs

To test the efficacy of the experimental drugs against *L. major* amastigotes, the infection rates (IR) and multiplication indices (MI) were determined as shown in Fig. 2. There was a general decrease in infection rate with increase in concentration of the treatments applied across the groups with significant difference between *T. vogelii* and the standard drugs (*p* = 0.01) whereas, infection of macrophages was not significantly affected when treated with Pentostam and Amphotericin B (*p* = 0.32). At the least concentration of 25 μg/ml, the macrophages were infected by amastigotes at 80% with *T. vogelii* treatment. At the same concentration Pentostam and Amphotericin B had 22 and 18% infection rates respectively. The infection rates reduced with increase in concentration with Amphotericin B and Pentostam falling from 18 to 2 and 22 to 3% respectively. The same trend was observed in *T. vogelii* from 80 to 20% when the concentration was increased to 200 µg/ml.

**Fig. 3:**
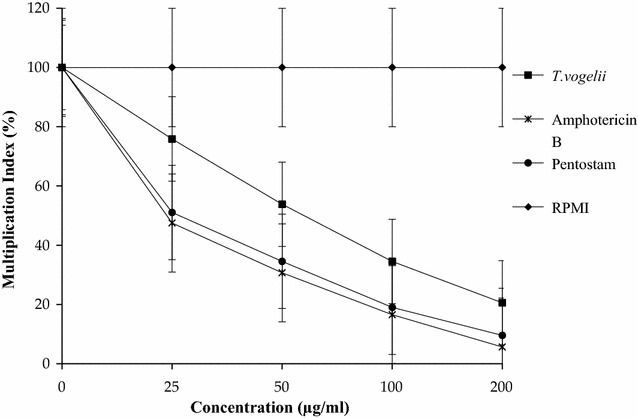
Multiplication indices (growth rates) of amastigotes in macrophages after treatment with the different test drugs

The Multiplication indices for *L. major* amastigotes in macrophages treated with 200 µg/ml of the test drugs were significantly different (*p* < 0.05); *T. vogelii* extract treatment showed a multiplication index of 20.57%, 5.65% for Amphotericin B and 9.56% for Pentostam. At a lower concentration of 25 µg/ml *L. major* amastigotes showed multiplication indices of 75.86% in *T. vogelii* extract, 47.5% in Amphotericin B and 51.07% in Pentostam. The multiplication indices are as shown in Fig. 3.

The optimal efficacy, IC_90_ and IC_50_ of the test drugs against amastigote forms of the parasites were significantly inhibited by the various test compounds (Table 2 describes). There were significant differences in the optimal efficacy of the test drugs (*p* = 0.0001). The optimal efficacy of the standard drugs was 91 and 98% for Amphotericin B and Pentostam respectively. *Tephrosia vogelii* achieved an optimal efficacy of 88.5% against amastigotes. There was a significant difference in the IC_50_ and IC_90_ of the test compounds (*p* < 0.05). The standard drugs were more effective against amastigote as compared to *T. vogelii* with Pentostam having an IC_50_ of 6.8 μg/ml, Amphotericin B with 5.8 μg/ml while that of *T. vogelii* being 9.2 μg/ml. There was significant (*p* = 0.001) difference in the IC_90_ with the lowest IC_90_ occurring in Pentostam followed by Amphotericin B then by *T. vogelii* with 16.8, 24.5 and 58.5 μg/ml respectively.

**Table 2:** Describes the optimal efficacy, IC_90_ and IC_50_ of the test drugs against amastigotes forms of the parasites

	Drugs			Test statistics	
	*T. vogelii*	Pentostam	Amphotericin B	F. value	p value
Optimal efficacy (%)	88.5	98	91	19.654	0.0001
Concentration at optimal efficacy (μg/ml)	40.1	24.6	36.2	9.012	0.0012
IC90	58.5	16.8	24.5	20.250	0.0120
IC50	9.2	6.8	5.8	12.612	0.001

### Effect of *T. vogelii* crude extracts on *L. major* lesion development in BALB/c mice

**Fig. 4:**
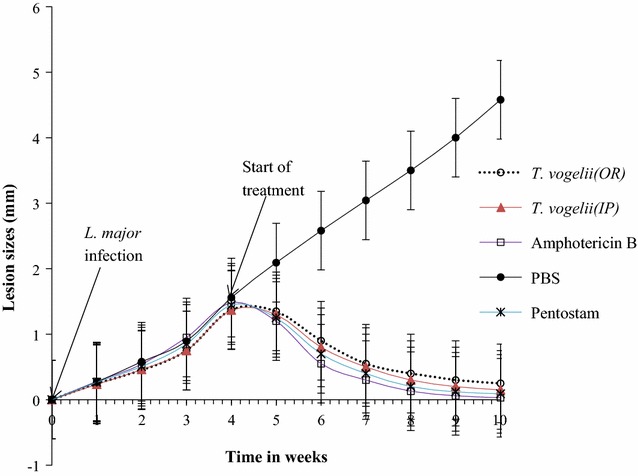
Lesion sizes of BALB/c mice at the start of infection, at the start of treatment and during the treatment with *T. vogelii*, PBS, Pentostam and Amphotericin B

The lesion sizes of BALB/c mice at the start of infection and in the course of treatment with the different test drugs are as shown in Fig. 4. There were no significant differences in development of lesion sizes in BALB/c mice during the first 4 weeks post-infection with *L. major* (*p* > 0.05). Between week 5 and 10, the differences in lesion sizes progression were subjected to repeated measure ANOVA, which indicated that there were significant differences in lesion sizes among different treatment groups (*p* = 0.004). The lesion sizes of the untreated control BALB/c mice increased steadily after infection. Smallest lesion sizes occurred in BALB/c mice treated with amphotericin B, which was slightly lower than BALB/c mice treated with Pentostam. BALB/c mice treated with *T. vogelii* intraperitoneally resulted insignificantly (p < 0.05) larger reduction of lesion sizes than the BALB/c mice treated orally with *T. vogelii*.

### Body weights, organs weight, organo-somatic indices and parasite loads in spleen and liver of BALB/c mice treated with different test drugs

There were significant differences in the weight of spleen, liver, spleno-somatic and hepato-somatic indices among treatment groups (*p* < 0.05). The untreated control group had significantly large spleen and liver weights alongside spleno-somatic and hepato-somatic indices accounting for the spleno and hepatomegaly observed. Pentostam and Amphotericin B treated groups had no significant difference between them producing the least spleen and liver weights and respective spleno-somatic and hepato-somatic indices. *Tephrosia vogelii* treatment produced an intermediate between the untreated control and the standard drugs treated groups. However, intraperitoneal administration of the test extract resulted in lower spleen/liver weights and organo-somatic indices than the oral administration mode (*p* < 0.05). Body weights, weight of spleen, liver and organo-somatic indices in BALB/c infected with *L. major* infection under various treatments are as shown in Table 3.

**Table 3:** Body weight, organ weight and organo-somatic indices in BALB/c mice after different treatments

Treatment	Body weights (g)	Weight of spleen (g)	Spleno-somatic index	Weight of liver (g)	Hepato-somatic index
*T. vogelii* (IP)	21.10 ± 0.52	0.17 ± 0.021^b^	0.85 ± 0.10^b^	0.18 ± 0.021^b^	0.86 ± 0.01^b^
*T. vogelii* (OR)	21.02 ± 1.00	0.21 ± 0.005^c^	0.99 ± 0.06^c^	0.20 ± 0.05^c^	0.98 ± 0.07^c^
Amphotericin B	21.05 ± 0.54	0.14 ± 0.010^a^	0.74 ± 0.05^a^	0.15 ± 0.015^a^	0.73 ± 0.05^a^
Pentostam	21.06 ± 0.58	0.15 ± 0.010^a^	0.75 ± 0.05^a^	0.16 ± 0.010^a^	0.75 ± 0.05^a^
PBS	21.06 ± 1.72	0.38 ± 0.014^d^	1.83 ± 0.21^d^	0.37 ± 0.014^d^	1.83 ± 0.51^d^
F	2.21	32.25	69.23	29.1498	65.72
Df	4	4	4	4	4
p	0.043	0.0001	0.0003	0.001	0.000

The means in the same column having the same superscript show no significant difference between them

**Fig. 5:**
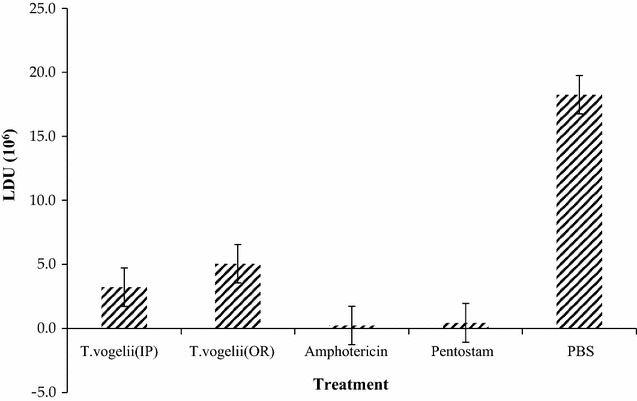
*L. major* LDU in spleens of BALB/c mice treated with different test drugs

The Leishman Donovan units (LDU) in the spleens of BALB/c mice subjected to oral and intraperitoneal administration of *T. vogelii* were significantly different (*p* = 0.0014). Treatment with *T. vogelii* intraperitoneally resulted in lower LDU (3.21 × 10^6^ than those observed when *T. vogelii* was administered orally 5.05 × 10^6^. Infected BALB/c mice treated with Amphotericin B and Pentostam resulted in the lowest LDU; 0.24 × 10^6^ and 0.45 × 10^6^ respectively but there was no significant difference between Pentostam and Amphotericin B treated groups. The LDU of *L. major* parasites in the spleens of BALB/c mice infected with *L. major* receiving *T. vogelii*, Amphotericin B, Pentostam and PBS are shown in Fig. 5.

**Fig. 6:**
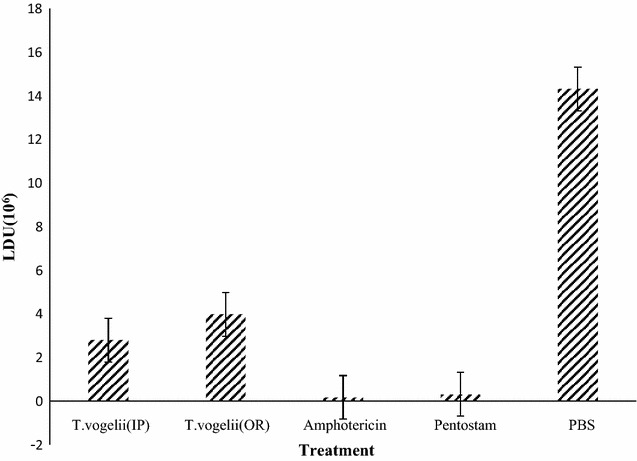
*L. major* LDU in liver of BALB/c mice treated with different test drugs

Intraperitoneal and oral administration of *T. vogelii* produced significantly different LDU trends (*p* = 0.002) in the liver. Pentostam and Amphotericin B resulted in significantly the lowest LDU; 0.18 × 10^6^ and 0.32 × 10^6^ respectively. However, there was no significant difference between Pentostam and Amphotericin B (*p* > 0.05) treated groups. The LDU of *L. major* parasite in the liver of BALB/c mice infected with *L. major* receiving *T. vogelii*, Amphotericin B, Pentostam and PBS treatments is shown in Fig. 6.

### Nitric oxide production in treated *L. major*-infected macrophages treated with different test drugs

**Fig. 7:**
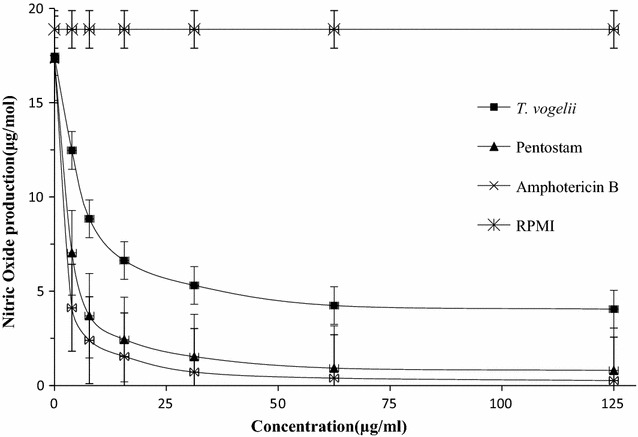
Nitric oxide production in the macrophages of *L. major* infected BALB/c mice and treated with different test drugs

There was significant difference in nitric oxide production in macrophages of BALB/c mice infected with *L. major* amastigotes and subjected to the different test drugs (*p* < 0.05). RPMI treatment produced the highest nitric oxide maintaining a consistent trend in all the concentrations. Amphotericin B treatment produced the lowest nitric oxide followed by Pentostam however, there was no significant difference between the two (*p* > 0.05). *Tephrosia vogelii* treatment produced higher amounts of nitric oxide compared to the standard drugs treated groups. Nitric oxide production in the macrophages of *L. major* infected BALB/c mice and treated with different test drugs is as shown in Fig. 7.

## Discussion

Despite existence of substantive chemotheraputants for different forms of leishmaniases there is also increasing evidence of parasite resistance to the conventional anti-parasitic therapies coupled with the prevailing traditional challenges of in accessibility, side effects and costs [50]. Moreover, vaccines against *Leishmania* species are still under development hence no vaccine exist yet [8]. Therefore, there is a need to search for alternatives and progressive validation of the same from fundamental research level to large scale trials where possible. Studies on medicinal plants and their chemotherapeutic activities against *Leishmania* species have been on the rise. These plants have demonstrated effectiveness against the parasite and making a contribution as alternative therapies [7]. In varying contexts, the benefit of scaling up chemotherapeutic options including using plant based products has been associated with the capacity to ensure decreased development of resistance by parasites [11, 51].

This study was carried out for the first time in the context to investigate activity of *T. vogelii* against *L. major*. The study was set to determine both the in vitro and the in vivo experimental outcomes. In the in vitro component, the cytotoxicity assay was set up to determine the toxicity levels of the test extract on vero cells. The cytotoxicity assay on the crude extracts of *T. vogelii* caused no significant adverse effects on the vero cells; this is further reflected in BALB/c mice peritoneal macrophages in vitro in the amastigote assay. The non-toxicity of *T. vogelii* is also reflected in the in vivo treatment using the extract, in which no mice died and none had a marked adverse degeneration. There was no marked change on weight, the appearance of the skin of the mice among other observable factors linked to physiological mechanism hence the plant extract was well tolerated. Compared to Amphotericin B and Pentostam, *T. vogelii* had less toxicity to vero cells and can lead to novel treatment for *L. major* infection.

*Tephrosia vogelii* extracts demonstrated remarkable in vitro anti-leishmanial activity against *L. major* promastigotes and amastigotes. In both assays, the activity of *T. vogelii* extracts against *L. major* showed that the plants contained some pharmacologically active substances that could prevent growth and proliferation of *L. major* promastigotes and amastigotes in the macrophage [52–57].

*Leishmania* parasite evades the host’s immune system by transforming from the flagellated promastigote to non-flagellated amastigote in macrophages of the mammalian host. This stage is critical for establishment of the infection [58]. This therefore needs a drug that will target the parasite in the amastigote form while inside the macrophages. A reduction in infection rates was evident following treatment with *T. vogelii* which is an indication that the extract suppressed either the establishment or the development process of *L. major* amastigotes in BALB/c peritoneal macrophages. The multiplication indices imply that the higher the concentrations the higher the inhibition rate of replication of amastigotes in the macrophages. The presented findings also indicated that *T. vogelii* extract caused marked reduction in splenomegaly and hepatomegaly (the parasite loads in the spleen and liver) of the mice during treatment period and reduced lesion development; suggesting that the extract indeed inhibited the growth of *L. major* parasite. *T. vogelii* given intraperitoneally gave better results than when given orally. This is due to the fact that intraperitoneal treatments usually act faster as there is enhanced uptake for the medicinal components than those given orally. However, the activity of the extract was lower than that of the standard drugs. This would possibly be attributed to the fact that *T. vogelii* was used in its crude form and isolation of active ingredients may improve its potency [17, 18].

Nitric Oxide is known to mediate macrophage cytotoxicity against microbes and tumor cells. It is usually upregulated in macrophages during *L. major* infection [57]. Apart from the normal nitric oxide produced by *Leishmania* infected macrophages naturally, the crude extract stimulated the macrophages in vitro to produce more nitric oxide for activity against the parasite indirectly. *T. vogelii* caused relatively higher induction of nitric oxide compared to the controls. This suggests that the plant extract employ the mechanism of nitric oxide cytolytic activity to eliminate the amastigotes which could be due to the different compounds present in *T. vogelii* extract.

There are some limitations associated with the presented finding. First, the in vivo treatment majorly focused on oral and intraperitoneal mode of administration hence other modes of treatment that is intravenous and subcutaneous should also be explored. To confirm on the non-toxic nature of the plant extracts, the effect that various factors such as the growth stage and maturity of the plant, regional variations (where the plant is growing) should be looked into. Leishmaniasis associated immunomodulatory markers were not analyzed in vivo. The evaluation can help give more insight on the validity of the extracts.

## Conclusions

Based on the findings of this study, *T. vogelii* extract exhibited anti-leishmanial activity with inhibitory action in both promastigotes and amastigotes with the activity and toxicity levels being compared to the standard drugs. The plant extract had significant activity when administered orally, hence suggesting can be administered both orally and intraperitoneally unlike the standard drugs that can only be administered through a specified mode. It was interesting to note that, the plant extract seemed to act in synergy with immunomodulation to wade the parasitic infection intracellular as observed through increased production of nitric oxide by the stimulated macrophages. Further studies should be done on isolating and testing individual active compounds which may give better anti-leishmanial activity and limit toxicity.

## Abbreviations

IC: inhibitory concentration
NTDs: neglected tropical diseases
THP: Traditional Health Practitioners
RPMI: Rosewell Park Memorial Institute 1640 Medium
KEMRI: Kenya Medical Research Institute
CTMRD: Centre for Traditional Medicine and Drug Research
FBS: Foetal Bovine Serum
MEM: Minimum Essential Medium
MTT: 3-4,5-dimethgylthiaol-2-yl-2, 5-diphenyltetrazolium bromide
DMSO: dimethylsulfoxide
IR: infection rate
MI: multiplication index
NO: nitric oxide
NO_2_: nitrite
LHFP: left hind footpad
PBS: phosphate buffered saline
LDU: Leishman-Donovan units
ANOVA: analysis of variance
